# 预后因素研究中常见的统计错误——偏倚

**DOI:** 10.3779/j.issn.1009-3419.2014.02.12

**Published:** 2014-02-20

**Authors:** Mithat Gönen, 北京大学肿瘤医院 付浩

**Affiliations:** Department of Epidemiology and Biostatistics, Memorial Sloan-Kettering Cancer Center, New York, New York

预后因素是患者诊治中非常重要的内容，大多数肿瘤患者的治疗决策取决于各种危险因素的存在与否。最突出的例子就是TNM（即肿瘤，淋巴结，远处转移）分期，但很多疾病都不能简单通过分期一个指标解决所有问题，而是要具体分析每个患者的综合预后因素。目前在判断胸腺瘤预后的临床研究中，一个主要的困难是缺乏实践指南，以至于到底哪些是预后因素尚不明确。本文叙述了临床实践中的一些问题并指出了临床研究中存在的各种偏倚，并试图提供解决方案，其中有许多方面在其它文献中作了详细讨论，但忽视了偏倚的存在。

毋庸置疑，可靠的预后因素具有重要的临床意义。若可以较为准确的预测病程，那么治疗方法的选择、患者随访的方法和咨询回答都会大大改善。因此，如其他疾病一样，胸腺瘤的所谓预后因素比比皆是^[1-7]^。其中，毫无疑问准确判断预后的能力在过去的二十年中有了跨越式发展。但是，仍只有极少数的标志物被纳入国际指南用于疾病的诊断、治疗和随访。本文将讨论这些所谓预后标志物最终失败的原因，并提供一些建议以尽量避免在未来的研究中类似情况的发生。

下面将列举几个在其它地方讨论过的例子，虽然不是胸腺瘤有关的内容^[8-13]^。其中一些例子来自于诊断因素的研究，因为在相关统计问题方面尤其是偏倚，诊断因素研究和预后因素研究中有很多共同之处。此外，诊断因素的例子通常具有简单易得的优势，这些例子均是大家所熟知的疾病。出于同样的原因，本文也提到一些关于肿瘤标志物研究和早期筛查的参考文献。

## 1 预后因素研究中的偏倚

偏倚是一个被过度使用的词，已很难给出一个确切的定义。本文中“偏倚”是指样本和总体之间的系统差别。例如，选择美国老年医保的高龄人群来研究甲状腺乳头状癌的预后，就存在重大偏倚，即甲状腺乳头状癌的中位年龄小于45岁，而所选样本是65岁及以上的老年人。这种差异被称为系统误差，不是因为抽样误差造成的，不会随样本量增加而克服。甲状腺癌例子存在所有说教比喻的通病：偏倚太明显了，掩盖了其他微小但重要的偏倚。举一个例子，假设一种“新的预后因子（NPF）”在某些恶性肿瘤中过度表达，多次体外研究之后一项胸腺瘤的回顾性临床研究报道其可能是潜在的预后因素，切除组织中表达NPF与不良预后相关（*P* < 0.05）。其他三项研究对此也进行了类似的回顾性分析，也报道了类似的结果。这三项研究中样本量最大的研究（比原研究还要大）确认了表达NPF者与非表达者之间存在生存差异。其他两项研究被认为是样本量小，未得出阳性结论。原研究者认为后两个研究样本量较小不具有足够的说服力并声称可以扩大样本量来验证，于是设计前瞻性研究先测定NPF的水平再探讨与结果的相关性，但他们失望地发现NPF的表达与生存没有显著相关性。

这是因为回顾性研究中存在的偏倚导致的吗？我们只能推测，但患者资料描述中提供了一个线索。回顾性研究利用的是存储的组织标本，完全有可能组织库中库存的标本来自较大的肿瘤可以满足不同研究的需要，而组织库中并未保存体积小的肿瘤。若果真如此，则样本（组织库中的肿瘤标本）和总体（所有的胸腺瘤）存在系统性误差。而前瞻性研究时需要纳入所有入组患者的标本，故偏倚较小或无偏倚。回顾性研究之间结论的不同可能源于这一偏倚。尽管只是假设，但该假设具有诸多的现实成分：大多数预后因素是经回顾性研究得出的，并可被一些（但并非全部）回顾性研究重复证实。但是大多数此类预后因素经不起标准更加严格的前瞻性研究的验证。事实上，所有回顾性研究都易出现偏倚。当然，前瞻性研究中也存在偏倚，但偏倚较小，且偏倚原因易于解释。

## 2 预后性研究中常见的偏倚类型

### 2.1 病例抽样偏倚

因病例选择而导致的偏倚称为患者选择偏倚或选择偏倚。该偏倚指入选患者组成的样本在疾病风险上偏向一极，要么太好，要么太坏，而不能代表该疾病的一般状况。癌胚抗原（carcino-embryonic antigen, CEA）是大家公认的结肠癌患者的预后标记物。Thomson等^[14]^的研究中，36例结肠癌患者有35例CEA升高，其敏感性为97%，这表明CEA有一定的诊断价值。但是10年后，情况就不理想了，Ⅰ期、Ⅱ期、Ⅲ期及Ⅳ期患者的敏感性分别为5%、25%、45%及65%^[15]^。这有力的推翻了将CEA作为诊断性标志物的用途。即使Ⅳ期患者，97%和65%的区别也很大，不能将此简单归因于一种因素。然而，有一种原因可以解释，那就是Thomson的研究对象明显不同于多数结肠癌患者，即选择偏倚。尽管PANS杂志并未交待这些患者的详细资料，很有可能这些病例均为局部晚期结肠癌患者。这就是选择偏倚最常见的来源。

另一常见的选择偏倚来源在前文中已经详细交待，在讨论分析NPF的可用组织时，库存肿瘤标本是体积较大的肿瘤而小者并未保留保本。从这个层面上看，CEA和NPF研究的偏倚来源是相同的，而从另一层面看又是不同的，在CEA研究中作者刻意选择极端晚期样本，而NPF研究中无此倾向，研究只利用组织库中所有可利用的标本。正是这一选择偏倚导致得出错误结论的危险，研究资料无其它选择并不意味着样本不存在偏倚。

### 2.2 对照选择偏倚

对照选择偏倚是另一种选择偏倚，是在病例对照研究中选择对照组时产生的^[16-18]^。理想情况下病例组和对照组之间的区别应只是研究因素不同，如暴露、疾病、治疗等。事实上，找到这样的对照组几乎是不可能的。如用血清肽筛查前列腺癌的例子就源于这种困难，病例组是25例经证实了的男性前列腺癌患者，对照组为健康男性，但是年龄小于40岁和血清中未检测到PSA^[19]^。一方面，选择对照组的条件或者依据并不像病例组那么严格，另一方面，对照组不可能进行活检以排除隐匿性恶性肿瘤，对照组因年龄小患前列腺癌的风险低，但是作者这么做又给两组带来另一个混杂因素：年龄。那么这时比较血清肽在病例组和对照组中的差异，不仅需要相关的专业知识，还需要这样一个假设：血清肽检测值与年龄没有关系。该例子也指出，选择不带偏倚的对照是不可能的。因此大多数研究倾向进行某种形式的匹配以减少偏倚，但不能完全消除偏倚。

### 2.3 双向数据挖掘的偏倚

这种偏倚更科学的名字应该是过拟合，但是数据双向挖掘清楚的概括了偏倚的根源，更适用说明文解释。数据双向挖掘是指用相同的数据进行多次关联分析而产生的偏倚。虽然数据双向挖掘的方式很多，但是在预后因素研究中体现尤为明显，当一个连续变量被不同界值在同一数据划分的时候就会产生。在发表的研究中很难找到该偏倚的详细例子，因此本文使用一个未经发表的例子。尽管该例子中的研究数据和设计方案都是真实的，但是本文对这些数据的分析仅用于说明双向数据挖掘偏倚。该例子提出的临床问题是，在术前化疗的患者中，PET扫描的摄取变化是否可以预测新辅助化疗后肿瘤的病理反应，其临床实用价值很明确，新辅助化疗后病理反应差的患者，即应改变治疗方案或早期手术，以把握治疗时机和减少新辅助治疗带来的副反应。研究者收集用于临床研究的数据如表 1和表 2所示。大多数SUV值大幅下降的患者有病理反应，反之亦然。事实上，仅有1例患者SUV值降幅大于35%而病理反应小于60%。表 2的统计结果非常令人鼓舞：SUV预测病理反应的敏感性为100%，特异性为90%。此外，估计阴性预测值为100%，也就是说，只要患者SUV值没有下降就意味着没有病理反应。但是大多数读者反对表 1和表 2的结论，他们指出该研究样本量过小和置信区间过宽。这当然是一个值得关注的问题，但是即使样本量足够大，仍然会有人持反对意见。见图 1所示的数据，首先我们暂时忽略虚线，可以看出SUV下降与治疗反应之间是存在一定关联的。图的左上部分几乎是空白的，一般来讲，SUV下降幅度越大对应的治疗反应也应该越大。根据大多数标准，SUV下降和治疗反应之间的关联系数是0.50是有意义的。这组数据的拟合线显示了一个固定的SUV值预测26%的治疗反应，而且SUV每下降10%，对应的治疗反应则增加6%。这样我们有理由相信PET扫描对最终治疗反应有一定的预测价值。然而，表 2夸大了数据分析结果。从图 1可以看出他们选择的界值（虚线）试图将不能分类的患者数量降到最低。这是选择界值常用的方式，图 1清楚的反应了表 2所列敏感性的大小取决于如何定界值。这些界值不能依靠一组单中心数据来选择，所以也不能评估敏感性，特异性和阳性阴性预测值。如表 3所示，即使界值很小的变动，都会对报道参数产生明显的影响，甚至可以改变研究结论。然而，如果用图 1代替表 1，表 2在一定程度上给SUV的预后价值评估奠定了基础。这个过度乐观的结论不仅是因为样本量小，而且还因为之后对数据的二次和三次挖掘造成的。

**1:** SUV下降与新辅助治疗后病理反应的关系 Correlation of a decrease in SUV after neoadjuvant therapy with pathologic response

	反应＞60%	反应≤60%	总数
SUV下降＞35%	3	1	4
SUV下降＜35%	0	9	9
总数	3	10	13
SUV:标化摄取值 注：本表已获得版权所有者© 2011 by the International Association for the Study of Lung Cancer复制许可。			

**2:** SUV下降＞35%预测新辅助治疗后病理反应的相关参数 Outcome Parameters for a Decrease in SUV of 35% in Predicting Pathological Response to Neoadjuvant Therapy

敏感性	特异性	阳性预测值	阴性预测值
100%	90%	75%	100%
SUV:标化摄取值 注：本表已获得版权所有者© 2011 by the International Association for the Study of Lung Cancer复制许可。			

**1:**
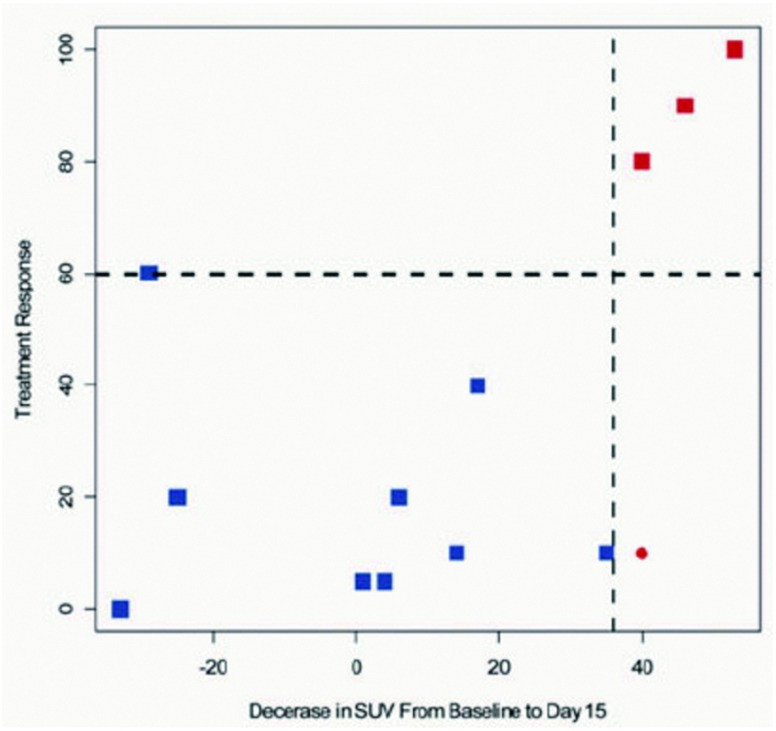
不同个体新辅助治疗后SUV变化与病理反应的关系 Individual data points of changes in SUV after neoadjuvant therapy and pathologic response

**3:** SUV界值改变对结果参数的影响 Effect of changes in thresholds on outcome parameters

界值	敏感性	特异性	阳性预测值	阴性预测值
SUV＞35%；病理反应＞60%	100%	90%	75%	100%
SUV＞30%；病理反应≥60%	75%	78%	60%	87%
SUV，标化摄取值。 注：本表已获得版权所有者© 2011 by the International Association for the Study of Lung Cancer复制许可。				

数据挖掘偏倚也可以在其他情况下出现。如果某人想得到一个预后值而对大量变量拟合一个模型，这个模型通常是对手头上的数据进行反复调整（这种情况下就存在过度拟合）。在同一组数据中评估模型的预测性能一定会导致乐观的结论^[20]^。虽然已经设计了一些统计方法来降低这些乐观的结论，但是这些统计方法并没有得到广泛应用，也没有被杂志编辑和审稿人所常规接受^[21, 22]^。即使这些方法不能完全消除因过度拟合造成的乐观结论，但是最终结论适用于单中心数据。

## 3 偏倚中的统计问题

须强调的是，通过巧妙的统计分析消除偏倚是极其困难的。理想情况下，一些统计模型可以减少偏倚，但代价是设定更多的假设，但是这些假设并不都是可以被证实的，其中一些问题在各种生物标志物研究指南和有影响的教科书中有讨论^[23, 24]^。比如，上述假设“新预后因子”的例子，假设在回顾性研究开始时，研究者怀疑他们的研究样本存在肿瘤大小的偏倚。除了在单因素分析中简单分析“新预后因子”与结局的相关外，他们可以选择把肿瘤大小和“新预后因子”放入多因素分析中，对肿瘤大小校正使结论更可靠。但是大多数类似的分析并没有注意模型所需要的假设。首先，需要选择一个合适的多因素回归模型，尽管Cox模型由于对假设要求宽松，在肿瘤学中已成为标准模型，但是仍然要求比例风险（PH）保持不变。只有某个因素和结局的相关性大小（通常用相对危险度表示）不随时间变化而变化PH才可以使用。但绝大多数的临床研究者并不能抓住这个假设的真正含义，而大多数数据分析者，可能由于缺少其他可替代的方法，并没有严格的审查就接受了这样一个假设。

除了PH模型还需要考虑函数形式。比如肿瘤大小，在模型里是否作为连续变量。尽管一般都假设肿瘤大小具有线性效应，即不管基础的大小是多少，大小每增加一个单位（比如1 cm）对应的影响效应增加是一样的，但往往实际模型更接近S型曲线，因为特别大或者特别小的肿瘤，模型曲线更平坦。大多数研究不管结论如何，都倾向采用线性模型，因为他们认为（或许乐观的认为）S型曲线的中间部位才有效，可以近似认为是直线。但是如果研究最初纳入的肿瘤标本体积比随机样本中的肿瘤大，则S曲线中对分析影响最大的部分应该是左边较为平坦的部分（即对应较小肿瘤的部分）。因此，一些研究者选择根据大小进行分组研究，而且分组研究结果更容易解读。如前文所述，分组也存在一些问题，即选择不同的界值对同一组数据进行分析，导致过度拟合（双向数据挖掘偏倚）。

本文列举了预后因素研究中一些常见的偏倚来源及其对结论可能的影响，但这些偏倚不是被首次发现和讨论，无数研究已进行过类似的探讨。但是，在许多发表的研究中仍然存在这些偏倚，而且未对这些偏倚的影响进行说明，这使得研究结果很难达到研究者的预期目标。严格来讲，这些偏倚并不是纯统计学问题，只是它们是在统计分析时被发现，而且统计学家试图通过统计模型来校正这些偏倚。但是在研究完成后处理偏倚是不合适的。最好的办法是在设计阶段就考虑周全，并让不同的成员，包括统计学家、病理学家、放射学家等参与讨论。虽然这可能会延缓数据搜集进度，但有利于结果的分析和解释，增强结论的可信度。

减少这些偏倚不仅仅是研究者的责任。编辑、审稿人和读者都应该严格遵循临床研究原则来要求高质量的研究报道。存在这样一个简单的问题，即某个因素何时可以被归为“预后的”因素。图 2显示了一个预后因素发生的经典过程。目前的做法是在A点标记预后因素，即在临床研究之后和验证之前。但这样做导致某些所谓的预后因素得不到验证或不能通过验证或只是部分验证，但仍被称为“预后因素”。建议在称某个因素为预后因素之前要进行仔细验证并考虑其临床实用价值。实际上，只有到B点后才能被称为预后因素，这样更具临床意义。虽然达到B点的因素远远比A点的少，但是A点的因素很多都是未经验证的假阳性预后因素，所以在B点我们并未漏评预后因素。

**2:**
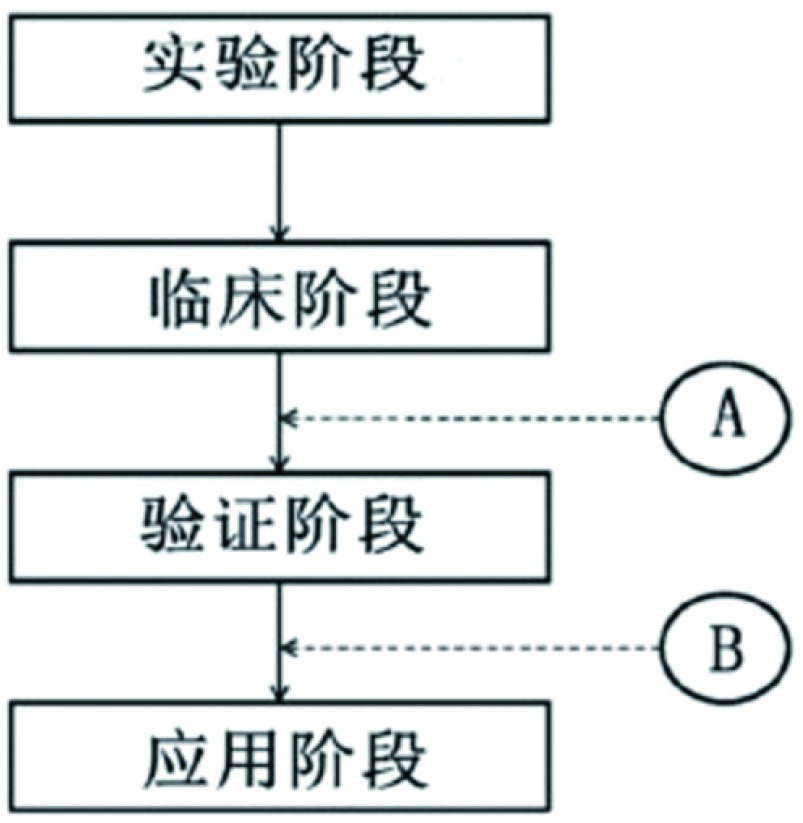
筛选和评估临床实用预后因素的流程 Phases in the identification and evaluation of a robust clinically applicable prognostic factor

总之，准确预测患者结局是非常有意义的，关键在于发现足够多的预后因素。但是我们的数据中存在很多固有偏倚，而且统计分析细节中也存在导致乐观结论的问题。本文指出了一些常见问题，以避免研究者和读者被误导。建议在研究中持谨慎态度，严格评估数据，对结果耐心验证。
